# Compliant Solid Polymer Electrolytes (SPEs) for Enhanced Anode-Electrolyte Interfacial Stability in All-Solid-State Lithium–Metal Batteries (LMBs)

**DOI:** 10.1021/acsapm.4c00806

**Published:** 2024-06-26

**Authors:** William R. Fullerton, Christopher Y. Li

**Affiliations:** Department of Materials Science and Engineering, Drexel University, Philadelphia, Pennsylvania 19104, United States

**Keywords:** solid polymer electrolytes, network solid polymer electrolytes, electrode−electrolyte interfaces, lithium−metal batteries, solid-state batteries

## Abstract

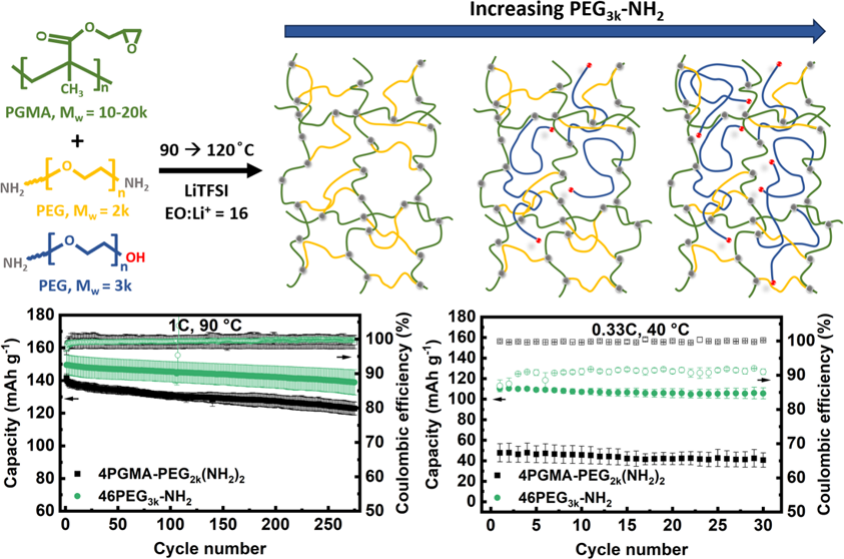

Practical application of high energy density lithium–metal batteries (LMBs) has remained elusive over the last several decades due to their unstable and dendritic electrodeposition behavior. Solid polymer electrolytes (SPEs) with sufficient elastic modulus have been shown to attenuate dendrite growth and extend cycle life. Among different polymer architectures, network SPEs have demonstrated promising overall performance in cells using lithium metal anodes. However, fine-tuning network structures to attain adequate lithium electrode interfacial contact and stable electrodeposition behavior at extended cycling remains a challenge. In this work, we designed a series of comb-chain cross-linker-based network SPEs with tunable compliance by introducing free dangling chains into the SPE network. These dangling chains were used to tune the SPE ionic conductivity, ductility, and compliance. Our results demonstrate that increasing network compliance and ductility improves anode-electrolyte interfacial adhesion and reduces voltage hysteresis. SPEs with 56.3 wt % free dangling chain content showed a high Coulombic efficiency of 93.4% and a symmetric cell cycle life 1.9× that of SPEs without free chains. Additionally, the improved anode compliance of these SPEs led to reduced anode-electrolyte interfacial resistance growth and greater capacity retention at 92.8% when cycled at 1C in Li|SPE|LiFePO_4_ half cells for 275 cycles.

## 1 Introduction

The lithium metal anode is considered one of the most promising routes for the commercialization of high energy density all-solid-state lithium–metal batteries (LMBs) due to its high theoretical capacity (3860 mAh g^–1^) and lowest standard electrode potential (−3.04 V vs SHE).^1−6^ However, when paired with current commercial liquid electrolytes, a host of problems associated with anode instability are known to arise. For example, lithium metal that reacts spontaneously with liquid electrolytes tends to produce a solid electrolyte interphase (SEI) on the lithium metal surface that is inhomogeneous in composition and mechanically brittle.^4,5,7,8^ An inhomogeneous SEI will promote heterogeneous nucleation and highly dendritic growth. Additionally, the brittle SEI makes it prone to cracking during charging and discharging, exposing fresh lithium to react with electrolyte and increasing SEI thickness. Furthermore, the high volatility of the liquid electrolyte poses a significant fire hazard that can facilitate thermal runaway when the cell fails via short circuit. These phenomena render the current use of commercial liquid electrolytes with a lithium metal anode untenable, which necessitates the search for alternative electrolyte solutions that can stabilize the lithium metal anode.

The use of solid polymer electrolytes (SPEs) as a battery separator can mediate many of the previously stated issues associated with liquid electrolytes.^1−6^ The advantages of using SPEs are their ability to (1) planarize dendrite growth and homogenize ion flux at the anode interface^4,9,10^ and (2) minimize SEI buildup from anode-electrolyte parasitic reactions.^3,6^ Additionally, they are low cost, easily processable, and lightweight, which makes commercialization and implementation in electric vehicles a reasonably achievable goal.^3,6,9,10^ Early physical modeling of lithium dendrite propagation by Monroe and Newman suggested that designing SPEs with a shear modulus twice that of lithium metal (∼6 GPa) should be sufficient to suppress dendrite propagation.^11,12^ Since then, solid polymer and inorganic electrolytes designed with high moduli such as polystyrene-*b*-poly(ethylene oxide) (SEO)-based SPEs,^13−17^ garnet-based,^18−21^ or sulfide-based^22−24^ electrolytes have displayed improved stability over liquid electrolytes and linear poly(ethylene oxide) (PEO)-based SPEs. However, these designs have displayed poor lithium electrode wetting which leads to interfacial instability.^16,25−27^ Later designs based on cross-linked elastomeric SPEs demonstrated greatly improved cycling stability compared to the aforementioned works, with elastic moduli on the order of 0.1–10 MPa, far less than the predicted 6 GPa.^28−33^ Additionally, solid electrolytes based on chemistries that yield more compliant and plastically deformable interfaces,^34−36^ and those with self-healing bonding,^37−39^ have displayed improved voltage hysteresis stability and increased cycle life compared to high modulus SEO or inorganic solid electrolytes. Recent work by our lab has also demonstrated that SPE mechanical toughness plays a critical role in slowing dendrite propagation and extending cycle life.^40^ These reports suggest that although adequate modulus is crucial to inhibit dendrite propagation, other SPE properties such as toughness and interfacial compliance are equally important in SPE design.

The present work is motivated by the hypothesis that for network SPEs, improving anode compliance should allow the SPE to adapt to the evolving lithium interface and facilitate more homogeneous ion flux. To this end, we utilized the comb-chain cross-linker-based network SPE (ConSPE) developed in our previous work as the model system since the comb-chain cross-linker has ultrahigh functionality: 142 epoxide groups for each chain of 20 kDa molar mass. The high functionality and fast cross-linking kinetics afforded using a comb-chain cross-linker allows for the synthesis of robust free-standing ultrathin (<20 μm) SPE films with homogeneous network structures. Thus, this platform enables the incorporation of large fractions of monofunctional poly(ethylene glycol) (PEG) into the network to increase free volume and compliance without losing the network property. Another advantage of introducing monofunctional PEG to tune the SPE mechanical property is that it bears the same chemistry as the bifunctional PEG that is used to form the network SPE. Therefore, any property changes upon introducing these free dangling chains can be attributed to a mechanical origin. All SPEs in this work display relatively high elastic modulus and toughness compared with previously reported SPE systems that have exhibited superior LMB performance.^28,29,32,33,41−44^ Interestingly, the SPE with the highest compliance exhibited the longest cycling life in both symmetric and asymmetric Li/Cu cells. By increasing SPE compliance, Li/Li symmetric cell cycle life is increased by 1.8× that of stiffer SPEs without dangling chains. Additionally, increasing SPE compliance showed a reduction in electrode-electrolyte interfacial resistance growth throughout cycling. Furthermore, the most compliant SPE architecture displayed a high Coulombic efficiency (CE) of 93.4% when employed in Li/Cu asymmetric cells. When implemented into Li|SPE|LiFePO_4_ half cells, this SPE displayed a high-capacity retention of 92.8% after 275 cycles and reduced anode-electrolyte interfacial resistance growth. The improved stability of this SPE design suggests that beyond a threshold modulus and toughness, increasing network SPE compliance is important for minimizing anode-electrolyte impedance and extending cycling life.

## 2 Experimental Section

### 2.1 Materials

Poly(glycidyl methacrylate) (PGMA, *M_n_* 10–20 kDa), poly(ethylene glycol) diamine (PEG, *M_n_* 2 kDa), poly(ethylene glycol) diamine (PEG, *M_n_* 6 kDa), *O*-(2-aminoethyl)polyethylene glycol (M-PEG, *M_n_* 3 kDa), lithium bis(trifluoromethane)sulfonimide (LiTFSI), tetrahydrofuran (THF), and dimethylformamide (DMF) were purchased from Sigma-Aldrich. Lithium foil, LiFePO_4_, Super P, and copper foil were purchased from MTI.

### 2.2 Sample Preparation

PGMA and LiTFSI salt were first placed in a vial with 1/3 vol % THF 2/3 vol % DMF at a total solid to solvent concentration of 25 mg mL^–1^. The solution was stirred in an oil bath at 60 °C for 3 h until fully dissolved. The PEG was then added to the solution with 2/3 vol % THF 1/3 vol % DMF at a total solid to solvent concentration of 160 mg mL^–1^. The solutions were cast onto a glass slide, and the samples were cured under vacuum at 90 °C for 24 h and 120 °C for an additional 12 h of post curing. After curing, samples were cut into 12 mm × 12 mm films then dried again for 16 h at 120 °C to remove moisture before transferring into the glovebox.

Sol fractions of the ConSPEs were determined by weighing the films before and after soaking in THF for 4 days then drying under vacuum at 120 °C for 24 h. The weight fraction of the salt in the network was subtracted out in order to leave only mass loss due to polymer chains unincorporated into the network.

LiFePO_4_ cathodes were prepared by mixing active material, super P, and 4PGMA–PEG_6k_(NH_2_)_2_ precursor with LiTFSI (EO/Li 16:1) at the weight ratio 56:14:30 in DMF followed by doctor blade coating onto aluminum foil and drying for 24 h at 40 °C. After solvent removal, the cathode film was placed between two stainless steel plates and c-clamped together. The cathode was then cured under this external pressure at 120 °C for 12 h under vacuum. After curing, the cathode calendared to 90–95% of its initial thickness by hot pressing at 90 °C for 30 min. Electrodes were then punched and dried under vacuum for an additional 16 h at 120 °C to remove moisture before transferring into the glovebox.

### 2.3 Electrochemical Cell Preparation

Symmetric cells were assembled using 750 μm thick lithium foil. Coin cells were assembled using a washer spring and 500 μm thick stainless steel spacer. SPEs tested during this experiment ranged from 70 to 100 μm in thickness. The cells were crimped under the same pressure to minimize any differences in cycling conditions. All cells were rested for 4 h at 90 °C prior to cycling. The cells were precycled at a current density of 0.03 mA cm^–2^ with an areal capacity of 0.12 mAh cm^–2^ for 6 cycles prior to transference number measurements or symmetric cell cycling.

Li|SPE|Cu cells were assembled using 750 μm thick lithium foil and acetic acid-treated copper foil. Coin cells were assembled using a washer spring and two 500 μm thick stainless steel spacers. SPEs tested during this experiment were approximately 70 μm in thickness. Coin cells were precycled at a current density of 0.05 mA cm^–2^ and capacity of 0.025 mAh cm^–2^ for 6 cycles prior to cycling.

Li|SPE|LFP cells were assembled using 750 μm thick lithium foil and an LFP cathode with a mass loading between 2.1 and 2.2 mg cm^–2^. Coin cells were assembled using a washer spring and two 500 μm thick stainless steel spacers. Coin cells were precycled for 3 cycles at 0.1C prior to cycling. For these mass loadings, this corresponds to a current density between 0.036 and 0.037 mA cm^–2^.

### 2.4 Sample Preparation of Electrochemical Cells for Post-Mortem Analysis

Cycled Li/Li symmetric cells used to obtain cross sectional scanning electron microscopy (SEM) images were first prepared by disassembling coin cells in the glovebox. After disassembly, the symmetric cells were cut in half at room temperature with a razor blade in order to expose the interior cross sections of the of the cells.

Cycled Li/Cu asymmetric cells used for X-ray photoelectron spectroscopy (XPS) analysis were prepared by first quenching the cells in liquid nitrogen in order to facilitate delamination between the SPE and lithium metal. The cells were then transferred into a glovebox and disassembled. SPEs were peeled off of lithium-plated Cu foil at a peeling angle of 180° to minimize the peeling force and encourage clean delamination between the SPE and lithium metal.

### 2.5 Thermal, Mechanical, and Electrochemical Characterization

Thermal measurements were taken on the DSC Q2000. Samples were heated from 20 to 110 °C at a rate of 10 °C min^–1^ then cooled down to −90 °C at a rate of 10 °C min then were heated to 110 °C at a rate of 10 °C min^–1^. All thermal properties were obtained from the second heating cycle.

Tensile measurements were made on the TA Instruments Discovery Hybrid Rheometer-3 (DHR-3) at 90 °C. Samples were cut into 15 mm × 5 mm rectangular films. Samples were kept at 90 °C temperature for at least 90 s prior to measurements. Samples were drawn at a constant tensile rate of 10 mm min^–1^. Young’s modulus was determined by taking the slope of the stress–strain curve in the linear elastic regime of the SPE. Tensile strength was determined by taking the stress before material fracture. Elongation at break was taken as the strain before material fracture. Toughness was determined by integrating the area under the stress–strain curve. These values were averaged from three separate tensile experiments to determine the mechanical properties of each material.

Impedance measurements used to determine ionic conductivity were taken on the Parstat 2273 potentiostat. Films were sandwiched between two blocking stainless steel electrodes for all sample measurements. The samples were heated from 20 to 100 °C and annealed at 100 °C for at least 30 min. Impedance measurements were made on the first cooling cycle from 90 to 30 °C. Samples were held at each temperature for at least 15 min prior to measurements. Impedance measurements were taken over the frequency range of 0.1 Hz–1 MHz with an applied potential of 20 mV. Bulk resistance values were determined by fitting the Randle circuit to the Nyquist plot to obtain the semicircle touchdown point. The resulting conductivities were calculated using the sample thickness *L* and the cross-sectional area *S*:
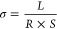


Electrochemical impedance and potentiostatic polarization measurements used to determine lithium-ion transference number (*t*_Li^+^_) were taken on the Gamry Interface Potentiostat/Galavostat/ZRA. Electrochemical impedance measurements were taken over the frequency range of 0.1 Hz–1 MHz before and after polarization to determine the interfacial resistance of the charge transfer reaction of the Li/Li symmetric cells. Polarization measurements were made under an applied potential of 20 mV for 8000 s and initial and steady state current values were taken at the beginning and end of the polarization experiment. Transference numbers were calculated using charge transfer resistance before polarization *R*_0_, charge transfer resistance after polarization *R*_ss_, initial current *I*_0_, steady state current *I*_ss_, and the potential drop Δ*V*
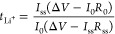


Linear sweep voltammetry was performed using the Gamry Interface Potentiostat/Galvanostatic/ZRA instrument using asymmetric Li|SPE|stainless steel cells. Scans were taken from 2.5 to 6 V at a rate of 0.1 mV s^–1^.

## 3 Results and Discussion

### 3.1 Synthesis and Characterization of ConSPEs

**Scheme 1:**
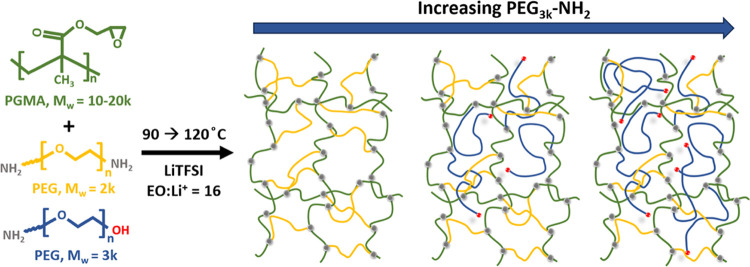
Monomer and Molecular Structure of ConSPEs

Scheme 1 shows the molecular architecture of the ConSPEs used in this study, where the samples are denoted as *x*PGMA*-*PEG*n*, in which *x* denotes the molar ratio of PGMA monomer/PEG(NH_2_)_2_, and *n* is the PEG molecular weight. When introducing monofunctional PEG, *M*_w_ = 3k (PEG_3k_-NH_2_) into the network, the PGMA monomer/amine ratio was held constant by replacing the moles of bifunctional PEG, *M*_w_ = 2k (PEG_2k_(NH_2_)_2_) removed from the network, with double the moles of PEG_3k_-NH_2_. These samples are denoted as *y*PEG_3k_-NH_2_ where *y* represents the mol % of PEG_3k_-NH_2_ out of the total PEG in the network. The network was formed from the reaction between the PGMA epoxide groups and amine end groups of the PEG chain. The resulting films were solid, flexible, and transparent, as seen in Figure S1, Table S1 lists the sol fractions of each ConSPE, which were determined by weighing the film before and after soaking in THF. Figure S2 shows the intact films after soaking and drying. The films all show a relatively low sol fraction (3.0–11.7 wt %), indicating efficient coupling reaction. Even for 46PEG_3k_-NH_2_, where over half of the PEG is monofunctional (56.3 wt %), the SPE displays a low sol fraction of 11.7 wt %. This indicates that most of the molecules are tethered into the network structure for all ConSPEs.

**Figure 1:**
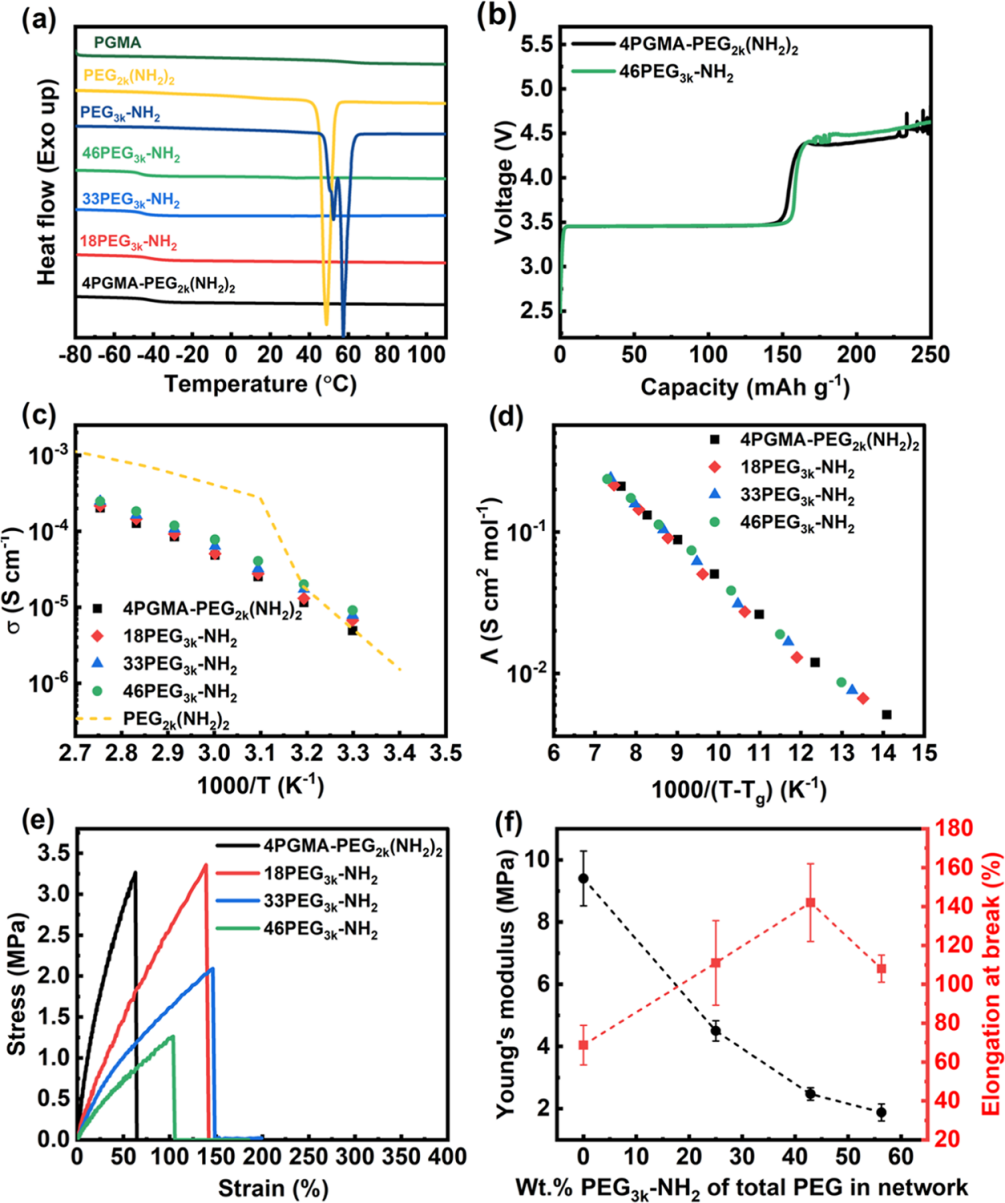
Thermal, electrochemical, transport, and mechanical properties of ConSPEs. (a) 2nd heating DSC thermograms of ConSPEs and salt-free monomers; (b) galvanostatic overcharging voltage traces; (c) temperature-dependent ionic conductivity; (d) molar ionic conductivity normalized by *T*_g_; (e) representative stress–strain traces determined through tensile measurements; and (f) the relationship between Young’s modulus, elongation at break, and percentage of PEG_3k_-NH_2_ incorporated into ConSPEs.

The DSC thermograms of the second heating scan of the ConSPEs and salt-free monomers can be seen in Figure 1a, while the thermal properties of the SPEs are recorded in Table 1. Even with the addition of more mobile free dangling chains, crystallinity (*X*_c_) is mostly suppressed as evidenced by the absence of crystal melting peaks from all of the thermograms except 46PEG_3k_-NH_2_. The trace melting peak in the 46PEG_3k_-NH_2_ sample corresponds to a small *X*_c_ of <1% for PEG in the SPE. The lack of crystallization is beneficial to achieving high ionic conductivity and is therefore desirable for device performance. The SPE glass transition temperatures are seen to decrease with increasing 46PEG_3k_-NH_2_ content, from −41.0 °C for 4PGMA–PEG_2k_(NH_2_)_2_ to −47.0 °C for 46PEG_3k_-NH_2_, which can be attributed to the reduced network connectivity and increased chain segmental mobility.

**Table 1:** Glass Transition Temperature *T*_g_, Ionic Conductivity, and Lithium-Ion Transference Number *t*_Li^+^_ of ConSPEs

			ionic conductivity (mS cm^–1^)			
SPE	EO in network (wt %)	*T*_g_ (°C)	30 °C	50 °C	90 °C	*t*_Li^+^_
4PGMA–PEG_2k_(NH_2_)_2_	77.3	–41.0	0.0049	0.025	0.20	0.15
18PEG_3k_-NH_2_	80.9	–44.0	0.0067	0.028	0.22	0.19
33PEG_3k_-NH_2_	83.1	–45.5	0.0078	0.032	0.25	0.17
46PEG_3k_-NH_2_	84.9	–47.0	0.0091	0.041	0.25	0.18

To determine the oxidative stability of the ConSPEs that would later be used in full cell cycling, galvanostatic overcharging experiments were performed at a rate of 15 mAh g^–1^(Figure 1b). The SPEs display an initial onset of oxidation at ∼4.4 V vs Li/Li^+^, which is substantially lower than the 5.3 V vs Li/Li^+^ limit determined through linear sweep voltammetry (Figure S3) for these SPEs. This highlights the importance of using such techniques for SPEs as the effective surface area and catalytic activity of inert electrodes such as stainless steel or platinum area quite different from that of actual battery cathode materials.^45,46^

Figure 1c,d displays the temperature-dependent ionic conductivity and *T*_g_ normalized molar conductivities of all SPEs, respectively, while Figure S4 shows a representative Nyquist plot. The lithium-ion transference numbers (Table 1 and Figure S5) range between 0.15 and 0.19, which is common for PEO-based SPEs.^6,47^ With increasing PEG_3k_-NH_2_ content, the conductivity increased as a result of two factors: (1) an increased number of ion carriers and (2) a decrease in the *T*_g_ that enhances segmental mobility. When normalizing molar conductivity by *T*_g_ (Figure 1d), the plots collapse onto one master trace, indicating the segmental mobility dictates differences in conductivity. Figure 1e displays the representative stress–strain curves, and Table S2 displays the determined mechanical properties. With increasing PEG3_k_-NH_2_ content, the Young’s modulus monotonically decreases from 9.4 MPa for 4PGMA–PEG_2k_(NH_2_)_2_ to 1.9 MPa for 46PEG_3k_-NH_2_ as displayed in Figure 1f. This can be attributed to the decrease in the network connectivity that contributes to its linear elastic response to applied deformation. The elongation at break reaches its maximum with 33PEG_3k_-NH_2_, then decreases with greater PEG_3k_-NH_2_ loading for 46PEG_3k_-NH_2_. This trend can be understood that when introducing PEG_3k_-NH_2_ into the network, the decreased cross-linking density generates higher molecular weight between cross-linking points, reducing the brittleness of the network and allowing for greater elongation before failure. Further increasing the PEG_3k_-NH_2_ content, i.e. low cross-linking degree, would reduce long-range interconnectivity, leading to earlier material fracture. This was observed in the case of 46PEG_3k_-NH_2_. Therefore, the toughness follows a bell curve-shaped trend, increasing with the addition of PEG_3k_-NH_2_ for 18PEG_3k_-NH_2_ and 33PEG_3k_-NH_2_ and decreasing to a minimum for 46PEG_3k_-NH_2_ due to loss of network connectivity as shown in Figure S6.

### 3.2 Evaluation of Dendrite Resistance and Electrochemical Cycling Stability

**Figure 2:**
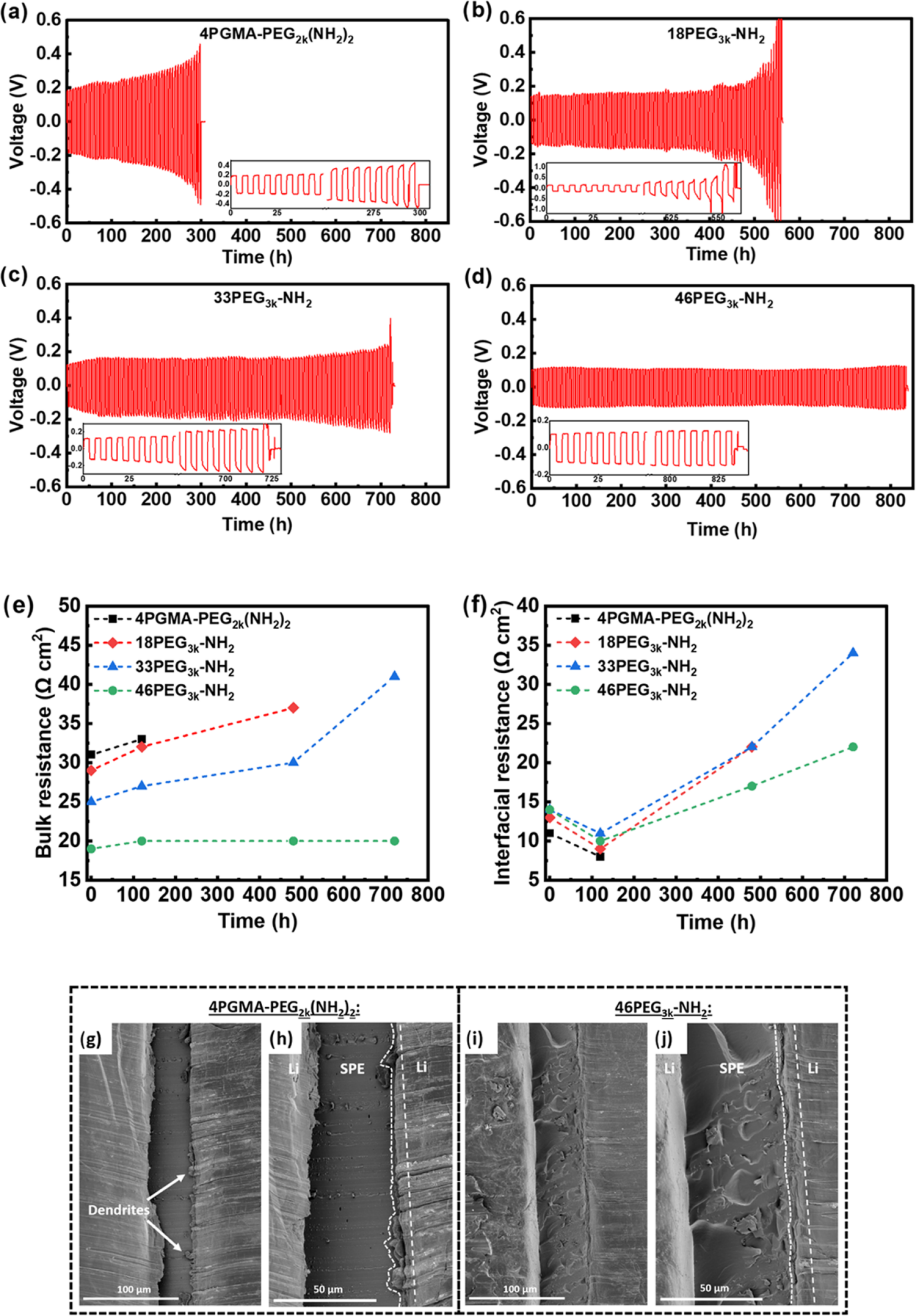
Stripping-plating tests for ConSPEs at current density of 0.5 mA cm^–2^ and areal capacity of 1.5 mAh cm^–2^ at 90 °C for (a) 4PGMA–PEG_2k_(NH_2_)_2_, (b) 18PEG_3k_-NH_2_, (c) 33PEG_3k_-NH_2_, and (d) 46PEG_3k_-NH_2_. (e) Bulk and (f) interfacial resistance vs cycling time obtained by EIS at various points in the symmetric cell cycling experiments. Cross sectional SEM images of Li/Li symmetric cells after 270 h of cycling for (g, h) 4PGMA–PEG_2k_(NH_2_)_2_ at low and high magnification, respectively, and (i, j) 46PEG_3k_-NH_2_ at low and high magnification, respectively.

To determine the ConSPEs’ resistance to dendrite propagation, galvanostatic stripping/plating experiments were employed by sandwiching the SPEs between two lithium electrodes. Figure 2 displays symmetric cell cycling voltage traces, revealing a reduction in voltage hysteresis growth and an increase in cycle life with increasing PEG_3k_-NH_2_ content in the network (Figure S7). Notably, 46PEG_3k_-NH_2_ exhibits the most stable voltage profile at extended cycling.

The voltage profiles of the stiffer 4PGMA–PEG_2k_(NH_2_)_2_ and 18PEG_3k_-NH_2_ exhibit the greatest voltage divergence prior to short circuit, which indicates an increase of diffusion-limited mass transport phenomena.^48,49^ The 4PGMA–PEG_2k_(NH_2_)_2_ cell experiences a voltage hysteresis growth of 274 mV on the charge and 307 mV on the discharge cycle from the first cycle until the last cycle prior to short circuit. The 46PEG_3k_-NH_2_ cell, however, only exhibits a growth of 21 mV on charge and 20 mV on discharge before short circuit. To better understand the individual kinetic contributions to voltage increases in the cells, electrochemical impedance spectroscopy (EIS) scans were taken at various points in the cycling experiment (Figure S8) and fit to the circuit model displayed in Figure S9. The resistance values obtained from the fitting are tabulated in Table S3. Figure 2e,f shows the evolution of bulk resistance and interfacial resistance with cycle number. Increases in bulk resistance in solid-state batteries have been associated with contact loss at the electrode–electrolyte interface due to surface roughening during cycling.^50−52^ The stable bulk resistance of the 46PEG_3k_-NH_2_ after 720 h of cycling suggests intimate contact is maintained between the lithium electrodes and electrolyte throughout cycling. Furthermore, the 46PEG_3k_-NH_2_ SPE displays the smallest growth in interfacial resistance (Figure 2f) at only 1.6× its initial value after 720 h. To better understand the different mechanisms of failure, cross sectional SEM images of the 4PGMA–PEG_2k_(NH_2_)_2_ (Figure 2g,h) and 46PEG_3k_-NH_2_ (Figure 2i,j) symmetric cells were taken after 270 h of cycling. The 4PGMA–PEG_2k_(NH_2_)_2_ cell exhibits clusters starting to protrude into the SPE layer, suggesting early dendrite formation on the plated electrode. Conversely, the 46PEG_3k_-NH_2_ SPE displays a more coherent electrode–electrolyte interface with more planar electrodeposited lithium morphology. It is worth noting that minor gaps can also been seen in the SPE-Li interface (top right in Figure 2j) of 46PEG_3k_-NH_2_, which is less significant compared with 4PGMA–PEG_2k_(NH_2_)_2_ (Figure 2h). The inhomogeneous appearance of the SPE in Figure 2j may be due to the sectioning process. The observed differences in the interfaces suggest that improved ionic conductivity and increased compliance allow the 46PEG_3k_-NH_2_ SPE to better conform to the evolving interface morphology and homogenize ion flux, delaying dendrite-induced short circuiting. Previous work by our group demonstrated that cycle life decreased when reducing Young’s modulus from ∼2.4–0.8 MPa by introducing oligomeric PEG, *M*_w_ = 250 into a POSS-4PEG_2k_-based network.^41^ Considering both these findings and our own results suggests that a modulus of ∼2 MPa probably provides the best balance between sufficient electrode contact and dendrite suppression ability. A modulus well below this will allow dendrites to easily propagate into the SPE while a higher modulus could lead to faster onset of electrode-electrolyte contact loss and interfacial instabilities that are detrimental to long-term cell performance.

**Figure 3:**
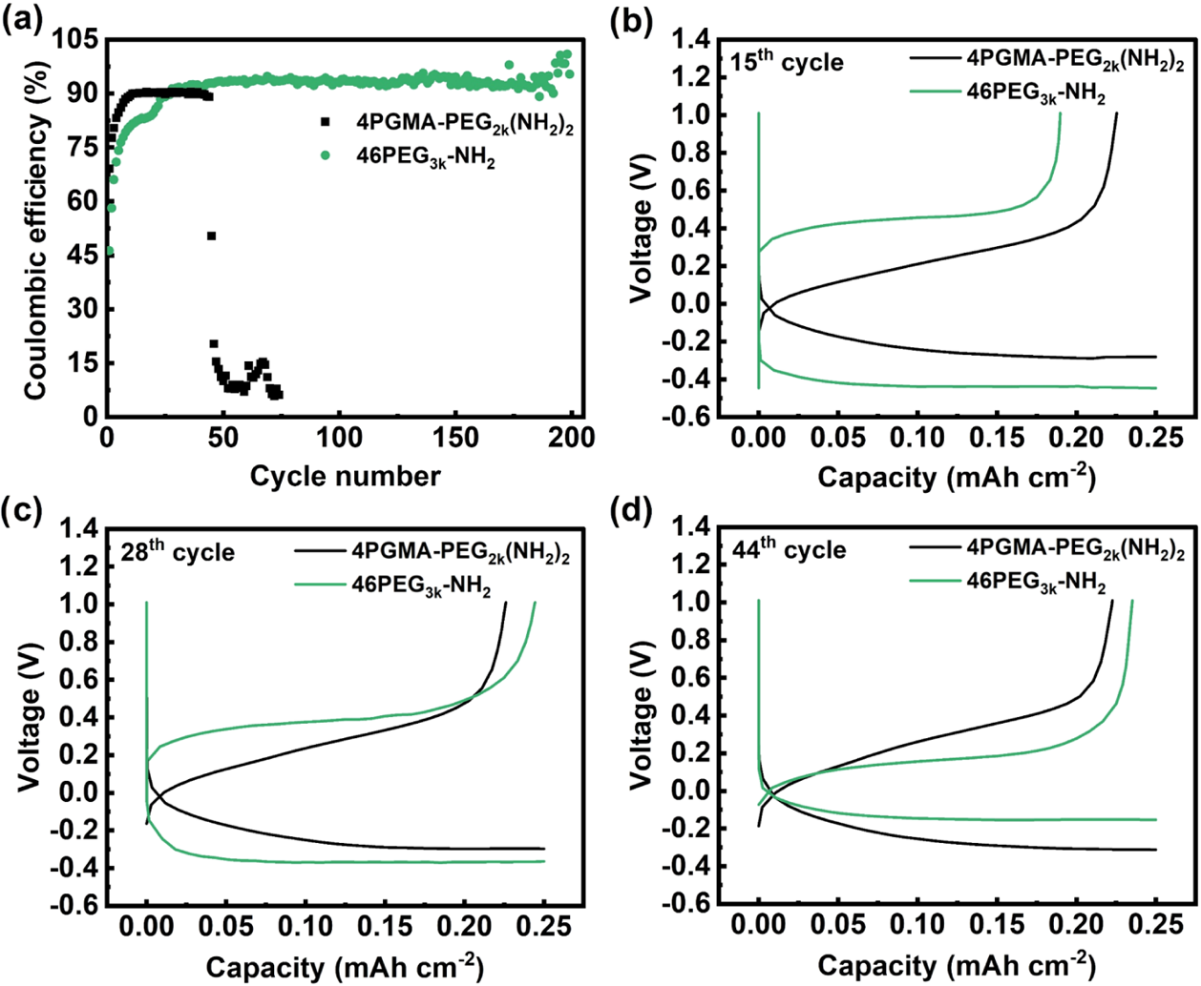
(a) CE of 4PGMA–PEG_2k_(NH_2_)_2_ and 46PEG_3k_-NH_2_ SPEs using a current density of 0.5 mA cm^–2^ and areal capacity of 0.25 mAh cm^–2^ at 90 °C. Plating and stripping profiles of the PGMA–PEG_2k_(NH_2_)_2_ and 46PEG_3k_-NH_2_ SPEs for the (b) 15th, (c) 28th, and (d) 44th cycles.

Ensuring uniform electrodeposit morphology and high CE using a copper current collector is essential for achieving the anode-free battery design necessary for high energy density lithium batteries. To this end, we used asymmetric Cu|SPE|Li cells to test the CE of our SPEs with Cu as the working electrode and Li as the counter and reference electrode. Lithium was plated onto the Cu current collector at a current density of 0.5 mA cm^–2^ and areal capacity of 0.25 mAh cm^–2^. The plated lithium was subsequently stripped from the Cu electrode until the voltage reached 1 V. The CE was determined by dividing the charge stripped from the Cu current collector by the charge plated. Figure 3a displays the CE for the 4PGMA–PEG_2k_(NH_2_)_2_ and 46PEG_3k_-NH_2_ SPEs. The 46PEG_3k_-NH_2_ SPE displays a CE that stabilized after 44 cycles and maintained a CE of 93.4 ± 0.8% until it reached 172 cycles, after which it began to fluctuate. The 4PGMA–PEG_2k_(NH_2_)_2_ SPE, on the other hand, exhibited a CE that quickly stabilized to 90.1 ± 0.3% after only 12 cycles, followed by a rapid drop after 44 cycles. This rapid drop in CE corresponds to a drop in the voltage Figure S10, indicating dendrite-induced short circuit. To confirm the consistency of this behavior, three additional cells with their CE profiles shown in Figure S11a were tested, all of which exhibited early short-circuit times, with an average occurrence after 36.3 ±21.5 cycles. Figure 3b–d shows the voltage profiles for both SPEs for the 15th, 28th, and 44th cycles which correspond to points in the cycling where the 46PEG_3k_-NH_2_ SPE exhibits a CE that is well below, slightly higher than, and at its highest point above the 4PGMA–PEG_2k_(NH_2_)_2_ SPE CE prior to its short circuit. The voltage hysteresis was taken as the offset of the charge and discharge profile halfway through each respective cycle. The 4PGMA–PEG_2k_(NH_2_)_2_ cell displayed a growth of voltage hysteresis from 521 to 568 to 593 mV on the 15th, 28th, and 44th cycles, respectively. The 46PEG_3k_-NH_2_ cell, however, displayed a reduction in voltage hysteresis from 904 to 757 to 322 mV on the 15th, 28th and 44th cycles, respectively, indicating the SEI formation process is still ongoing in these early cycles. The C 1s and O 1s of XPS scans taken after cells cycled for 38 cycles (Figure S12) displayed in Figure S13 show higher signal from the C–O peaks after etching of the lithium surface for 46PEG_3k_-NH_2_, which intimates that more PEO was reduced into its SEI than 4PGMA–PEG_2k_(NH_2_)_2_. This is likely due to the larger sol fraction of 46PEG_3k_-NH_2_ (11.7 wt %) compared to 4PGMA–PEG_2k_(NH_2_)_2_ (3 wt %). These excess untethered PEG molecules would be prone to migration to and reduction at the lithium anode interface. Greater irreversible reduction of PEG in the 46PEG_3k_-NH_2_ cell would also justify the slower stabilization process compared to 4PGMA–PEG_2k_(NH_2_)_2_. We posit that the polymer-rich SEI improves contact between the SPE and the lithium-plated Cu electrode, which better accommodates the volume changes during cycling that have been shown to exacerbate electrode-electrolyte delamination and accelerate short circuiting.^27,53−55^ The improved contact, along with the higher ionic conductivity and improved interfacial stability, is believed to account for the increased CE of the 46PEG_3k_-NH_2_ cell.

### 3.3 Demonstration of Li|SPE|LiFePO_4_ Full Cell Performance

**Figure 4:**
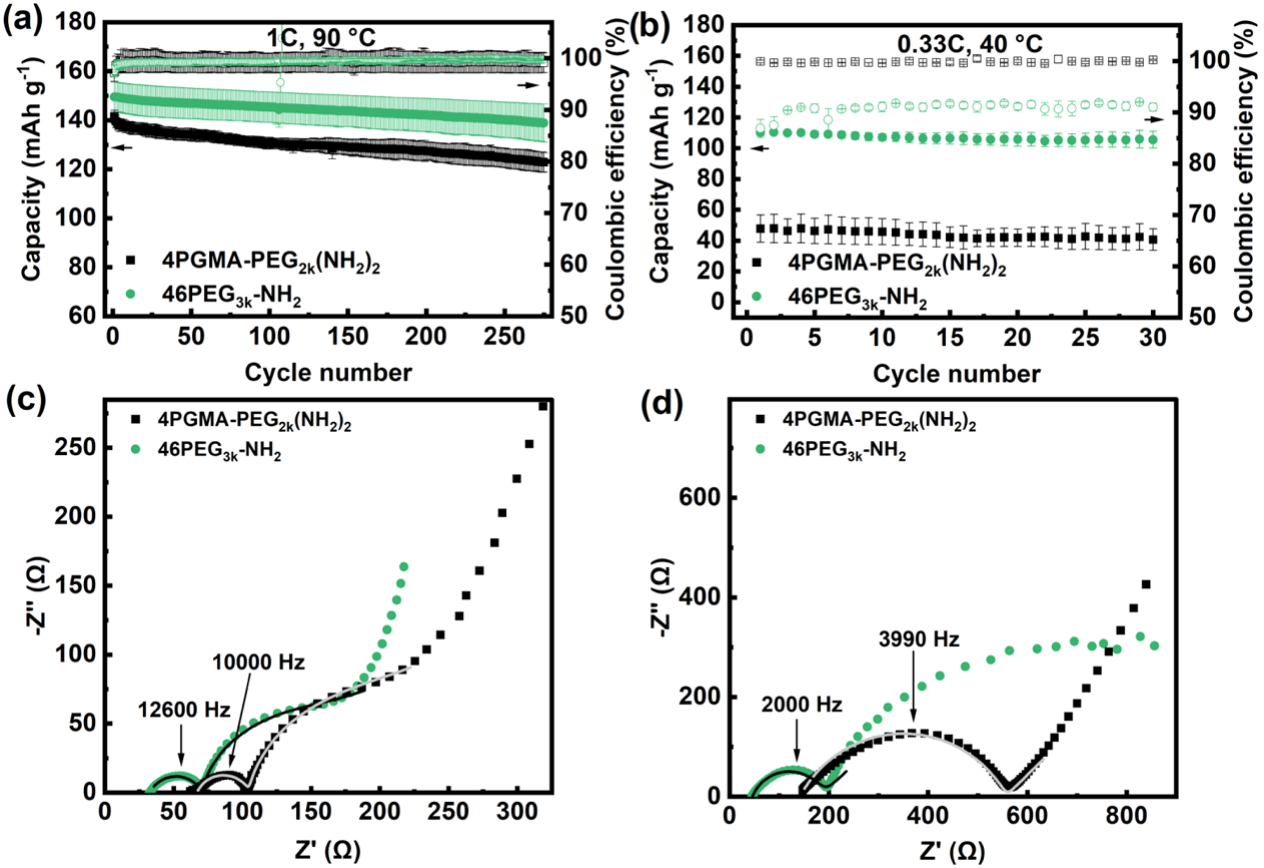
(a) Discharge capacity and Coulombic efficiency of 4PGMA–PEG_2k_(NH_2_)_2_ and 46PEG_3k_-NH_2_ at 1C and 90 °C and (b) at 0.33C and 40 °C. EIS scans (c) before and (d) after cycling of 4PGMA–PEG_2k_(NH_2_)_2_ and 46PEG_3k_-NH_2_ at 1C and 90 °C for 275 cycles.

To assess the impact of SPE molecular design on battery performance, we selected the 4PGMA–PEG_2k_(NH_2_)_2_ and 46PEG_3k_-NH_2_ SPEs to be paired with lithium iron phosphate (LFP) composite cathodes. In this work, we used 35 μm thick SPE films and composite cathodes with 4PGMA–PEG_6k_(NH_2_)_2_ binder (Figure S14a,b), as this molecular structure has demonstrated high ionic conductivity and superb mechanical toughness in our previous study.^32^Figure 4a,b displays the discharge capacity and CE of an average of two cells cycled at a rate of 1C at 90 °C and a rate of 0.33C at 40 °C. The mass loading between 2.1 and 2.2 mg cm^–2^ used in these cells corresponds to current densities of 0.36–0.37 mA cm^–2^ and 0.12 mA cm^–2^, respectively. At a rate of 1C and 90 °C, the 46PEG_3k_-NH_2_ cell exhibited a discharge capacity of 149.5 ± 6.0 mAh g^–1^, whereas the 4PGMA–PEG_2k_(NH_2_)_2_ cell only reached a capacity of 141.5 ± 2.6 mAh g^–1^. At 40 °C, the disparity in achievable capacity becomes significantly more pronouced. While the 46PEG_3k_-NH_2_ cell demonstrates a capacity of 110.3 ± 2.1 mAh g^–1^ at 0.33C, the 4PGMA–PEG_2k_(NH_2_)_2_ cell managed less than half of that, achieving a capacity of only 48 ± 8.8 mAh g^–1^. The higher achievable capacity of the 46PEG_3k_-NH_2_ SPE is likely attributed to the combined effects of higher ionic conductivity and compliance, which would enable it to conform well to the electrode interface and enhance ionic transport to the LFP particles. At lower temperature, these properties prove to play a much more consequential role in capacity utilization as evidenced by the severe overvoltage generated in the 4PGMA–PEG_2k_-(NH_2_)_2_ cell (Figure S15). At 90 °C and 1C, the 46PEG_3k_-NH_2_ cell exhibits a capacity retention of 92.8 ± 1.4% whereas the 4PGMA–PEG_2k_(NH_2_)_2_ cell displays a capacity retention of 86.8 ± 1.1% after 275 cycles (Figure 4a). This corresponds to a capacity loss of ∼0.03% per cycle for 46PEG_3k_-NH_2_, which is lower than most previously reported SPE systems cycled at equal or lower C-rates than in this study (Table S4).^32,42,56−61^

EIS scans were taken before and after cycling one cell for 275 cycles (Figure S16) and are shown in Figure 4c,d. EIS data before cycling was fitted to the equivalent circuit model displayed in Figure S17 and the resistance values are tabulated in Table S5. This model has previously been used for solid-state batteries where the semicircle at high frequency represents anode-electrolyte resistance and the semicircle at low frequency represents cathode-electrolyte resistance.^62,63^ Because no low frequency semicircle was observed in the 4PGMA–PEG_2k_(NH_2_)_2_ cell after cycling, the EIS data was fitted to the circuit model shown in Figure S18 in order to obtain bulk electrolyte and anode-electrolyte interfacial resistances shown in Table S6. The 46PEG_3k_-NH_2_ cell exhibited a bulk and interfacial resistance growth of 1.3× and 3.6× its original resistance values, respectively. The 4PGMA–PEG_2k_(NH_2_)_2_ cell, on the other hand, displayed a much larger growth of bulk and interfacial resistance at 2.2× and 10.5×, respectively. This suggests that the high stiffness of the 4PGMA–PEG_2k_(NH_2_)_2_ SPE accelerates contact loss and impedance growth at the lithium-electrode interface during cycling. The enhanced stability of the 46PEG_3k_-NH_2_ SPE in Li|SPE|LiFePO_4_ half-cell demonstrates the direct correlation between electrolyte compliance and device performance, highlighting its importance in SPE design.

## 4 Conclusions

In this work, a series of comb-chain cross-linker-based ConSPEs were designed to better understand the correlation between SPE network structure and LMB device performance. The ultrahigh functionality of the comb-chain cross-linker allowed us to introduce a large fraction of free dangling chains into the network without sacrificing the network property. Our results demonstrated that the more mechanically compliant SPEs displayed improved anode-electrolyte adhesion and cycle life compared to its higher modulus counterpart. SPEs with the largest addition of PEG_3k_-NH_2_ into the network structure displayed a 1.9× increase in symmetric cell cycle life with smoother lithium electrode morphology after 270 h of cycling. These SPEs also showed a higher Coulombic efficiency of 93.4% and longer cycle life when employed in Li–Cu cells. Li|SPE|LiFePO_4_ half cells utilizing SPEs with this molecular design displayed a higher capacity retention at 92.8% after 275 cycles and smaller anode-electrolyte impedance growth throughout cycling at 1C. Our results further demonstrated that the network structure is of crucial importance in SPE design for future LMBs.

## Supporting Information Available

Digital photos of SPE films; EIS characterization and fitting results; voltage profiles for Li/Cu asymmetric plating/stripping; Coulombic efficient of Li/Cu cells; XPS results; comparison with previously reported SPEs (PDF)
