# Supplementary material for: Compliant Solid Polymer Electrolytes (SPEs) for Enhanced Anode-Electrolyte Interfacial Stability in All-Solid-State Lithium–Metal Batteries (LMBs)
Source: ACS Appl Polym Mater. 2024 Jun 26;6(13):7468–77. doi: 10.1021/acsapm.4c00806 (PMC11250032; doi:10.1021/acsapm.4c00806)
Supplement: Supplementary file 1 — ap4c00806_si_001.pdf [file ap4c00806_si_001.pdf]

Supporting information for:

Compliant Solid Polymer Electrolytes (SPEs) For Enhanced Anode-Electrolyte Interfacial Stability in All-Solid-State Lithium-Metal Batteries (LMBs)

*William R. Fullerton and Christopher Y. Li**

Department of Materials Science and Engineering, Drexel University, Philadelphia, PA 19104, USA

KEYWORDS: solid polymer electrolytes, network solid polymer electrolytes, electrode-electrolyte interfaces, lithium-metal batteries, solid-state batteries

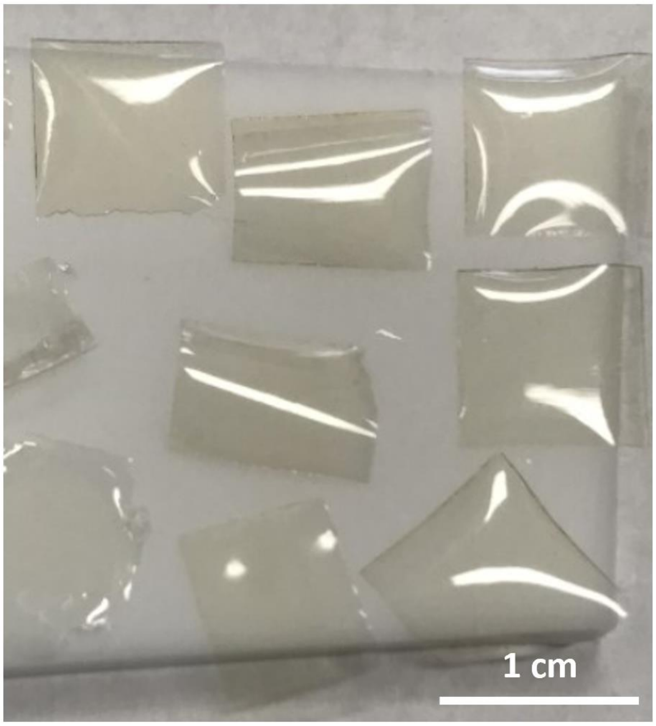

Figure S1. Photograph of 46PEG$_{3k}$-NH_2 SPE membranes.

Table S1. Electrolyte composition by weight percentage.

SPE	PGMA	$PEG_{2k}(NH_2)_2$	PEG_{3k}-NH_2	LiTFSI	Sol fraction
4PGMA-$PEG_{2k}(NH_2)_2$	16.9	59.6	-	23.5	3.0
18PEG_{3k}-NH_2	14.5	49.5	15.3	24.3	5.8
33PEG_{3k}-NH_2	12.7	35.7	26.8	24.8	7.5
46PEG_{3k}-NH_2	11.3	27.8	35.7	25.2	11.7

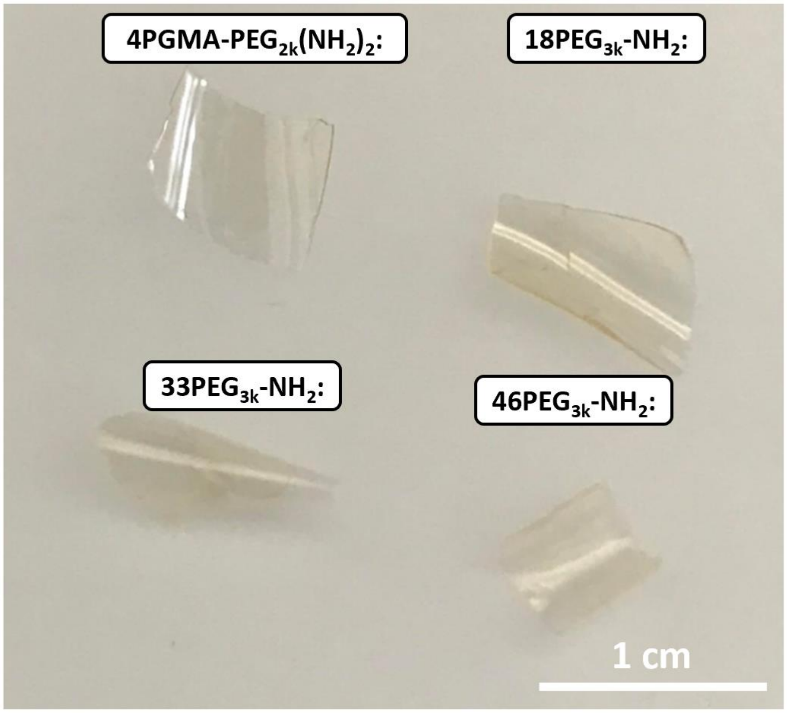

Figure S2. Photograph of ConSPEs used to determine sol fraction after soaking in THF and drying.

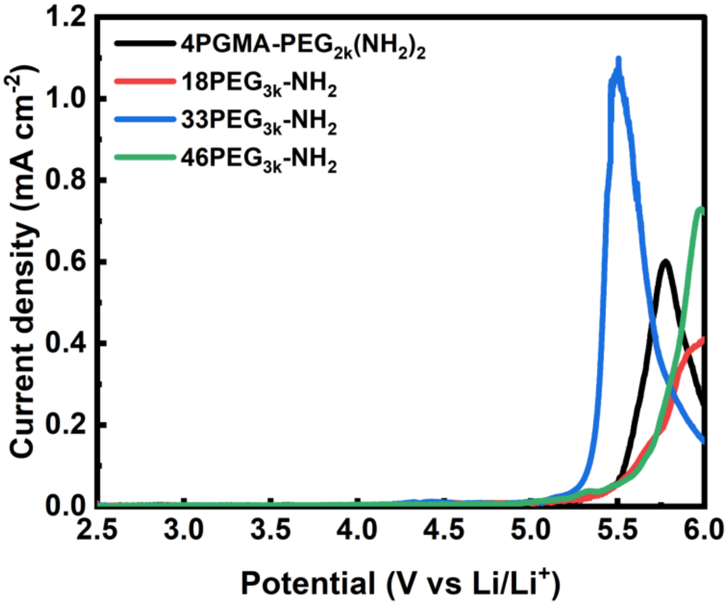

Figure S3. Linear sweep voltammetry traces for ConSPEs at scan rate of 0.1 mV s^{-1}.

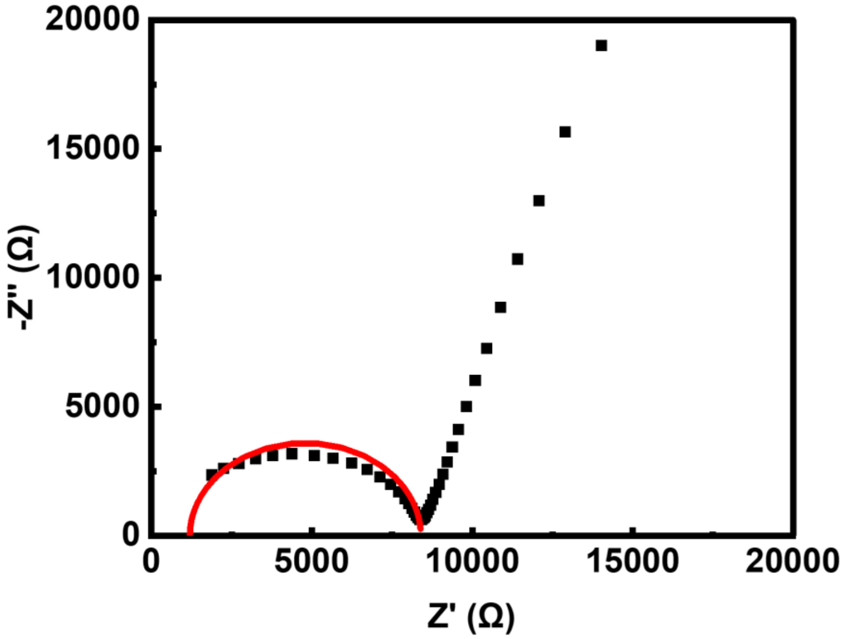

Figure S4. Representative Nyquist trace for stainless steel|4PGMA-PEG$_{2k}$(NH$_2$)$_2$|stainless steel cell at 30 °C. The impedance data was fit to an equivalent Randles circuit to determine the ionic conductivity.

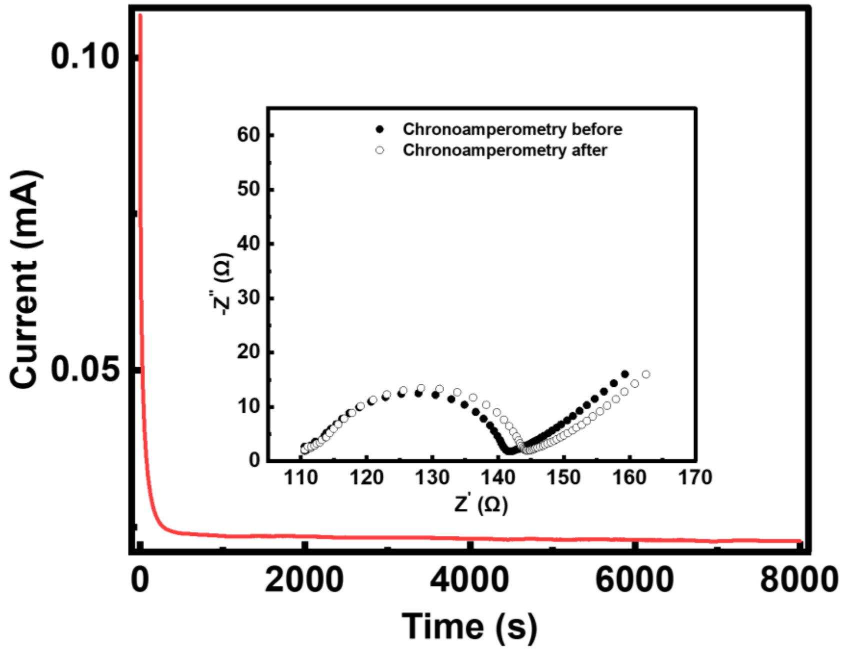

Figure S5. Representative chronoamperometry profiles and the impedance spectra before and after chronoamperometry test used to measure transference number for the symmetrical lithium cell 4PGMA-PEG$_{2k}$(NH$_2$)$_2$ at 90 °C.

Table S2. Mechanical properties of ConSPEs.

SPE	Young's modulus (MPa)	Tensile strength (MPa)	Elongation-at-break (%)	Toughness (M J m^{-3})
4PGMA-$PEG_{2k}(NH_2)_2$	9.40±0.88	3.27±0.21	69±10	1.38±0.29
$18PEG_{3k}$-NH_2	4.50±0.33	2.70±0.47	112±22	1.78±0.58
$33PEG_{3k}$-NH_2	2.47±0.20	2.06±0.17	142±20	1.68±0.21
$46PEG_{3k}$-NH_2	1.88±0.27	1.34±0.14	109±7	0.82±0.14

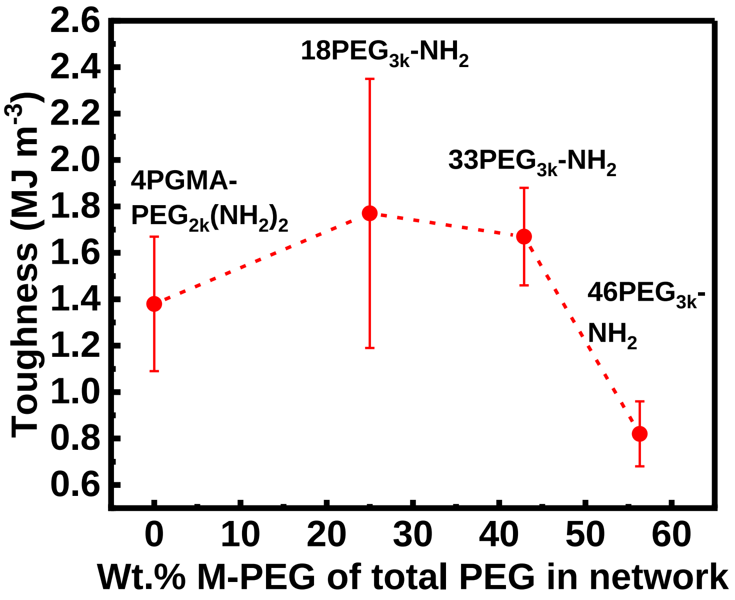

Figure S6. Toughness vs wt.% PEG_{3k}-NH_2 of total PEG in network for ConSPEs.

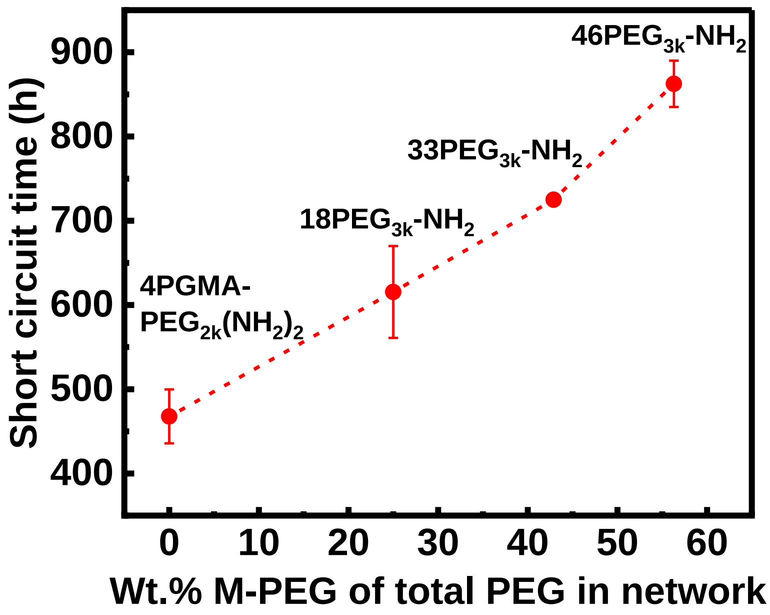

Figure S7. Plot of the short circuit time of ConSPEs vs. the percentage of PEG_{3k}-NH_2 out of total PEG in the network for Li/Li symmetric cells at a current density of 0.5 mA cm^{-2} and areal capacity of 1.5 mAh cm^{-2}.

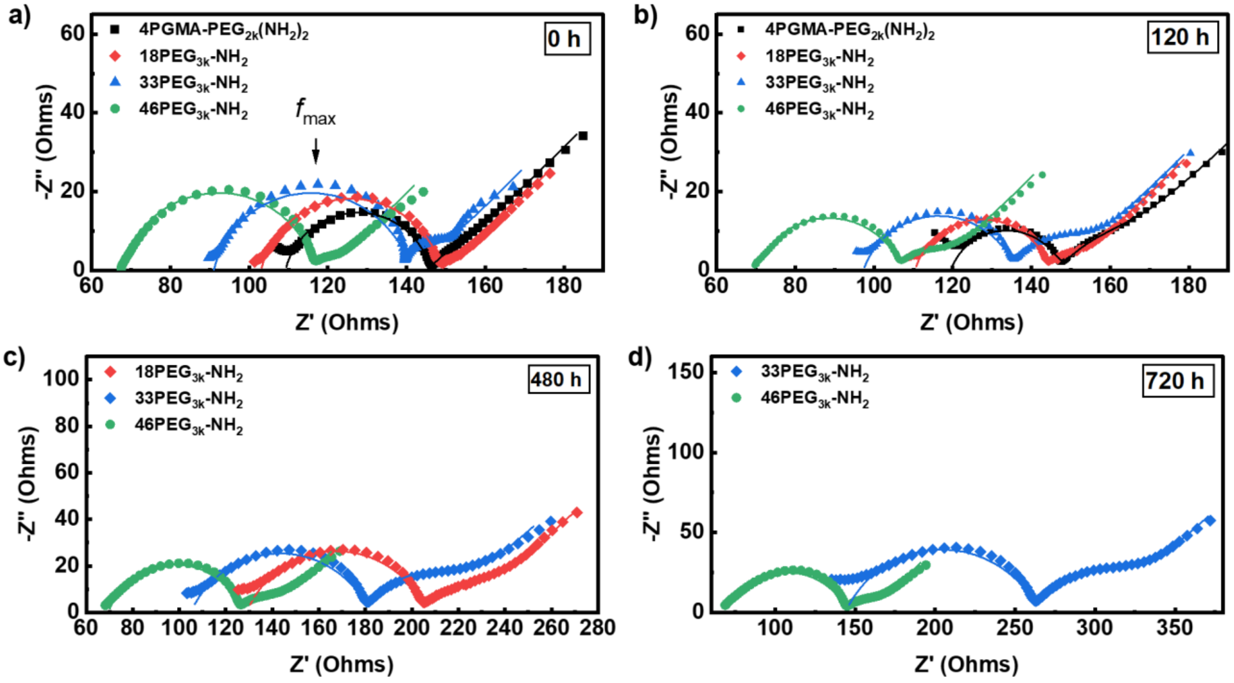

Figure S8. Electrical impedance spectroscopy (EIS) scans for ConSPE Li/Li symmetric cells (a) before; (b) after 120 hours; (c) after 480 hours and (c) after 720 hours of cycling at a current density of 0.5 mA cm^{-2} and areal capacity of 1.5 mAh cm^{-2}.

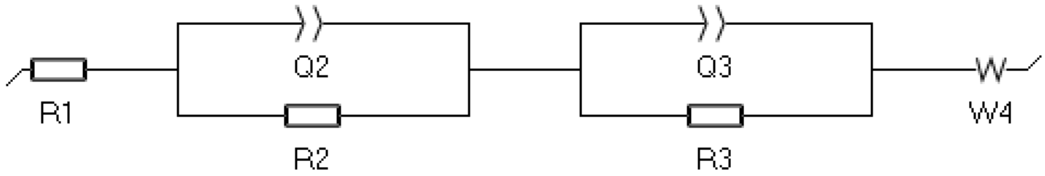

Figure S9. Equivalent circuit model used to fit EIS spectra taken throughout the symmetric cell cycling experiments. R_i , Q_i, and W_i represent resistive, constant phase and semi-infinite Warburg elements, respectively. R1 and R2 are taken to represent the bulk electrolyte and electrode-electrolyte resistances, respectively. The addition of R3, Q3 and W4 elements were introduced to improve the fit in the lower frequency region.

Table S3. Interfacial and bulk resistances obtained through fitting EIS data taken throughout the symmetric cell cycling experiments.

Cyle	SPE	Bulk resistance (Ω cm^2)	Interfacial resistance (Ω cm^2)	Maximum frequency (Hz)
Post Pre-charge	4PGMA-$PEG_{2k}(NH_2)_2$	31	11	12600
	$18PEG_{3k}$-NH_2	29	13	7928
	$33PEG_{3k}$-NH_2	25	14	7928
	$46PEG_{3k}$-NH_2	19	14	7928
Post 120 hours	4PGMA-$PEG_{2k}(NH_2)_2$	33	8	15880
	$18PEG_{3k}$-NH_2	32	9	12600
	$33PEG_{3k}$-NH_2	27	11	12600
	$46PEG_{3k}$-NH_2	20	10	12600
Post 480 hours	4PGMA-$PEG_{2k}(NH_2)_2$	-	-	-
	$18PEG_{3k}$-NH_2	37	22	15880
	$33PEG_{3k}$-NH_2	30	22	12600
	$46PEG_{3k}$-NH_2	20	17	12600
Post 720 hours	4PGMA-$PEG_{2k}(NH_2)_2$	-	-	-
	$18PEG_{3k}$-NH_2	-	-	-
	$33PEG_{3k}$-NH_2	41	34	12600
	$46PEG_{3k}$-NH_2	20	22	12600

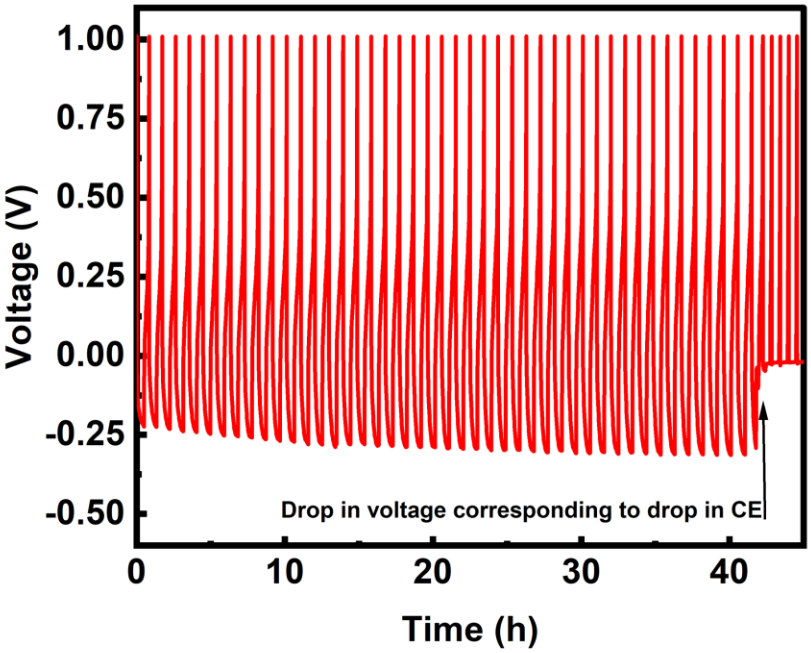

Figure S10. Voltage profiles for Li/Cu asymmetric plating/stripping experiments to determine CE 4PGMA-PEG$_{2k}$(NH$_2$)$_2$.

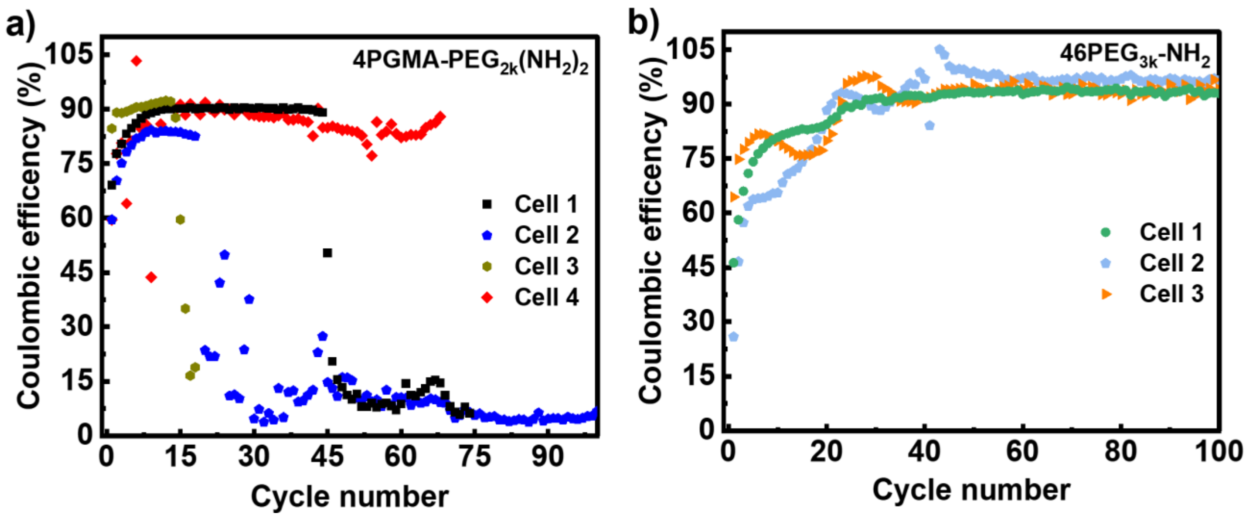

Figure S11. Coulombic efficiency of Li/Cu asymmetric cells for (a) 4PGMA-$PEG_{2k}(NH_2)_2$ and (b) $46PEG_{3k}$-NH_2 SPEs.

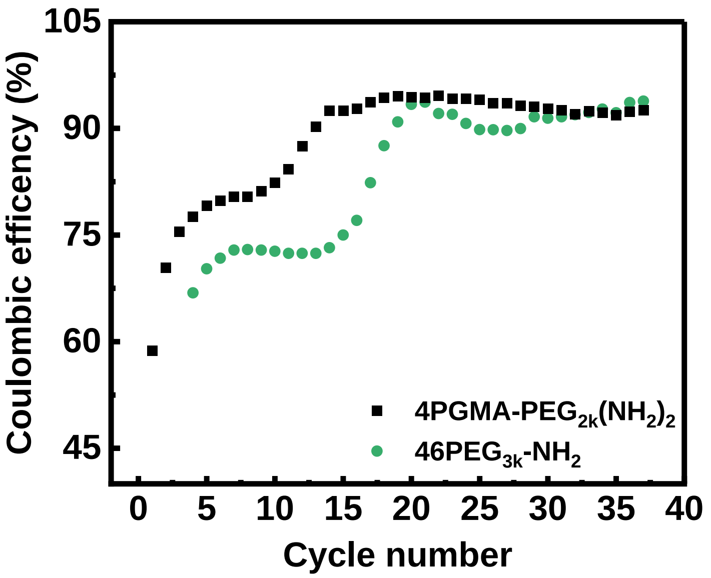

Figure S12. Coulombic efficiency of Li/Cu asymmetric cells cycled 38 times and disassembled for use in XPS characterization.

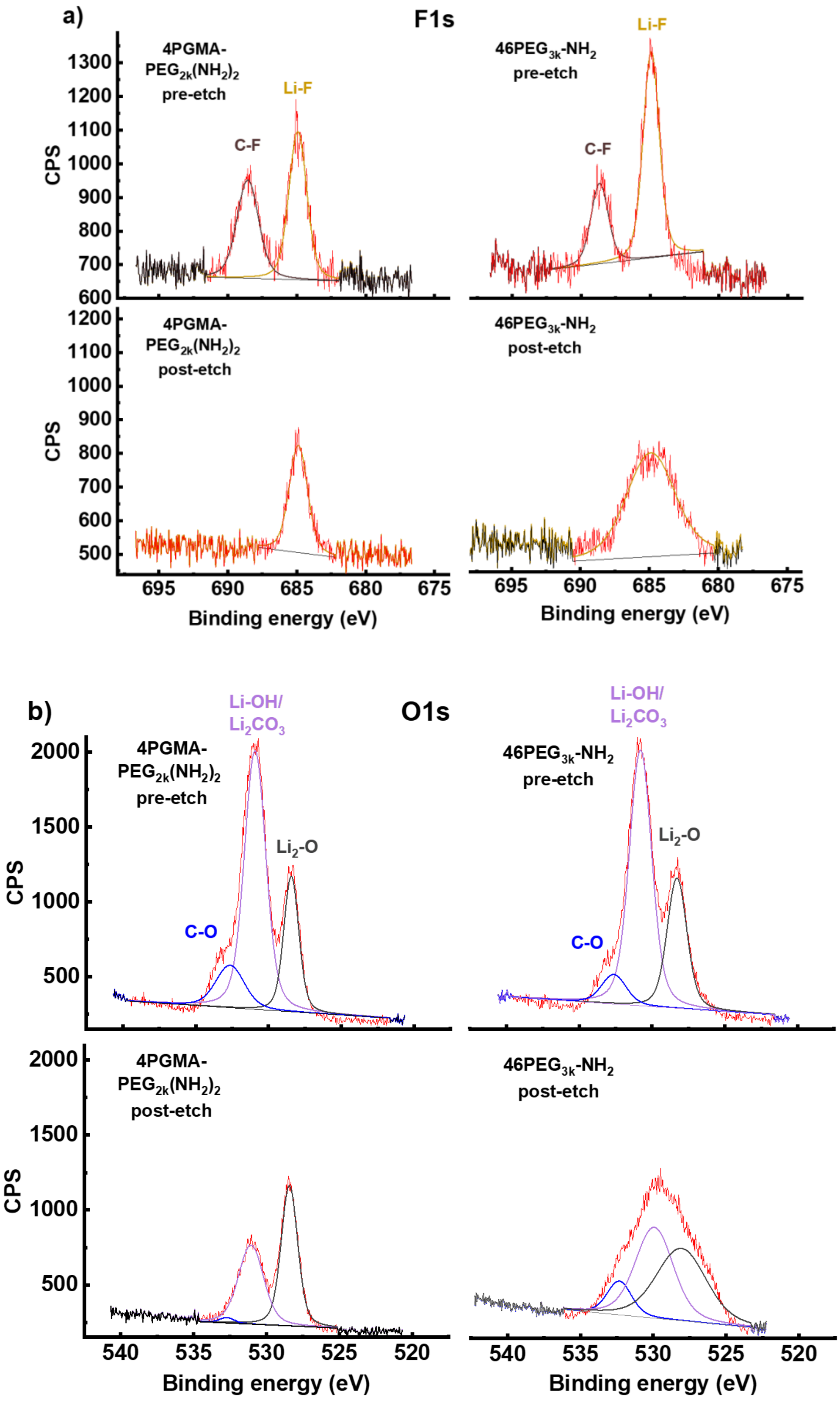
a)
F1s
4PGMA-PEG2k(NH2)2 pre-etch
Li-F
C-F
46PEG3k-NH2 pre-etch
Li-F
C-F
4PGMA-PEG2k(NH2)2 post-etch
46PEG3k-NH2 post-etch
CPS
Binding energy (eV)
b)
O1s
Li-OH/ Li2CO3
4PGMA-PEG2k(NH2)2 pre-etch
Li2-O
C-O
46PEG3k-NH2 pre-etch
Li-OH/ Li2CO3
Li2-O
C-O
4PGMA-PEG2k(NH2)2 post-etch
46PEG3k-NH2 post-etch
CPS
Binding energy (eV)

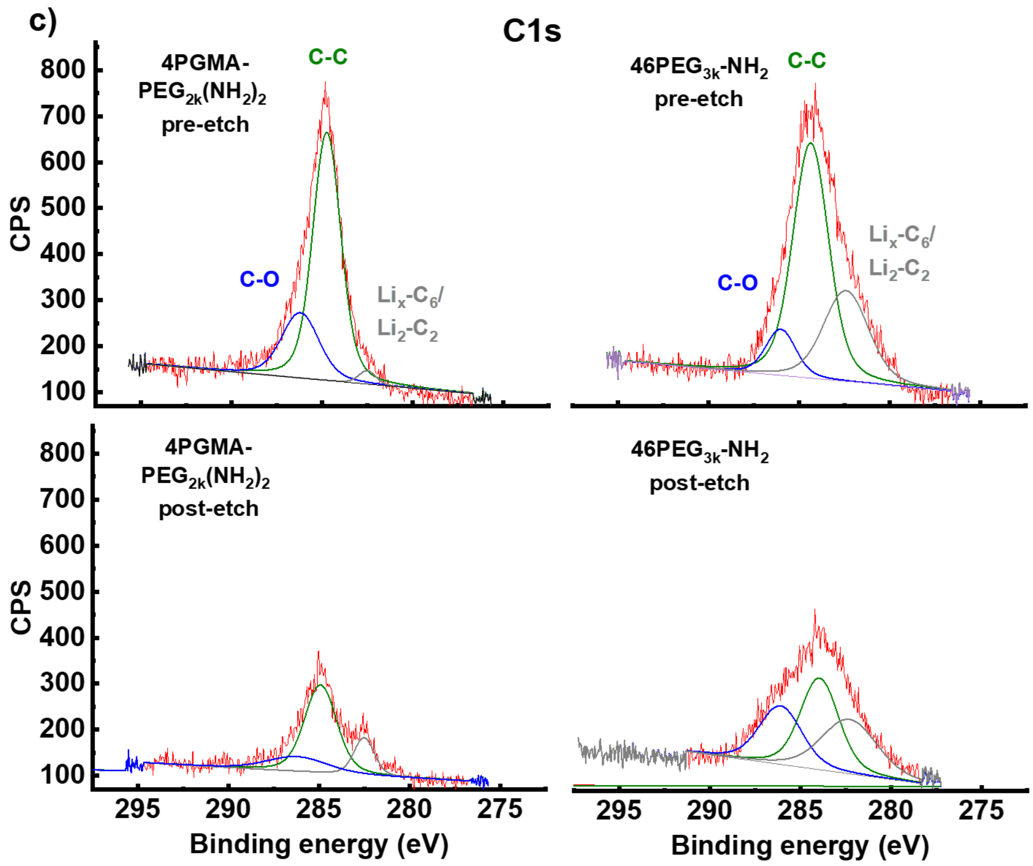

Figure S13. XPS spectra of cycled lithium metal surface in Li|SPE|Cu cells after 38 cycles at a current density of 0.5 mA cm^{-2} and areal capacity of 0.25 mAh cm^{-2}. (a) F1s (b) O1s and (c) C1s spectra for 4PGMA-$PEG_{2k}(NH_2)_2$ and 46PEG_{3k}-NH_2 prior to and after 1 min of etching with 2 kV Ar ion gun.

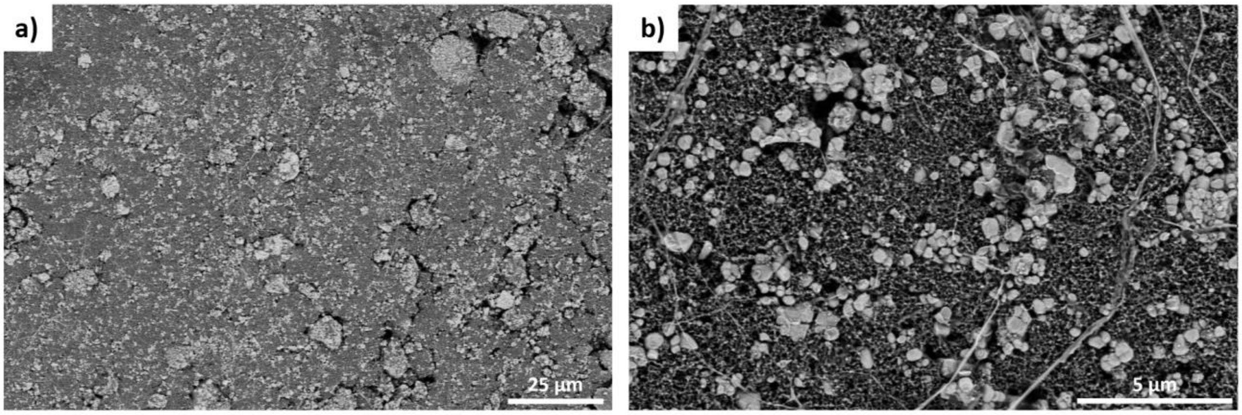

Figure S14. a) Low and b) high-magnification images of a $LiFPO_4$ composite cathode.

Table S4. Comparison of 46PEG$_{3k}$-NH$_2$ electrochemical performance to previously reported SPEs in literature (work published after 2019).

SPE	Temperature (°C)	Symmetric cell	Li-Cu coulombic efficiency	Li|SPE|LiFePO$_4$ cell cycling parameters: Mass loading (mg cm^{-2})	C-rate & Capacity (mAh g^{-1})	Capacity retention	Ref.
**This work**	**90**	**0.5 mA cm^{-2}, 1.5 mAh cm^{-2} 860 h**	**0.5 mA cm^{-2}, 0.25 mAh cm^{-2} 93.4%, 172 cycles**	**2.1-2.2**	**1C, 149.5**	**92.8%, 275 cycles**	
PEO-grafted-PCL polyrotaxanes/ LiTFSI	60	0.1 mA cm^{-2}, 0.1 mAh cm^{-2} 600+ h	-	1.5	1C, 117*	94%, 170 cycles*	1
Single-ion P(SSPSILi-altMA)+PEO	80	0.01 mA cm^{-2}, 0.04 mAh cm^{-2}, 1200+ h	-	2.2-3	0.1C, 117.5*	91.4% 300 cycles*	2
POSS-4PEG$_{2k}$ PIL5%-IPN/LiTFSI	90	1 mA cm^{-2}, 3 mAh cm^{-2} 808 h	-	2-3	0.5C, 150.4*	92%, 50 cycles*	3
PEO/LiTFSI PE matrix	60	0.1 mA cm^{-2}, 0.1 mAh cm^{-2} 1500 h	-	3	1C, 146	66%, 500 cycles	4
POSS-4PEG$_{2k}$-PPC IPN9-10PPC/ LiTFSI	90	1.5 mA cm^{-2}, 1.5 mAh cm^{-2} 271 h	-	2-3	0.2C, 149	92.7%, 200 cycles	5
4PGMA-PEG$_{6k}$/LiTFSI	90	2 mA cm^{-2}, 2 mAh cm^{-2} 703 h	-	2-3	1C, 139	86.4%, 200 cycles	6
PEGMEA /LiTFSI PMMA-PS matrix	60	0.2 mA cm^{-2}, 0.1 mAh cm^{-2} 1500 + h	-	-	1C, 165	76.4%, 1000 cycles	7
PEO/LiTFSI PAN matrix	60	0.5 mA cm^{-2}, 0.25 mAh cm^{-2} 300+ h	-	1.5	0.5C, 145.7	70%, 500 cycles	8
PVDF/LiTFSI	25	0.5 mA cm^{-2}, 0.5 mAh cm^{-2} 80 h	0.1 mA cm^{-2}, 0.2 mAh cm^{-2} 94%, 80 cycles	-	-	-	9

PEGMEA-Ga-LLZO[b]	RT	0.5 mA cm^{-2}, 0.25 mA cm^{-2} 100+ h	-	-	-	98.5%, 100 cycles	10
PEO/α-aluminum fluoride[b]	60	0.2 mA cm^{-2}, 0.2 mAh cm^{-2} 560+ h	-	2	0.3C, 167.4	84.5%, 500 cycles	11
PAN-SiO_2/LiTFSI[b]	RT, 80	0.3 mA cm^{-2} 2000+ h RT	-	-	3C, 165.8	94.9%, 300 cycles 80 °C	12
PEO/LiTFSI LLTO matrix[b]	60	-	-	1	1C, 149.2*	97.2%, 150 cycles*	13
Poly-DOL/ LiTFSI+ $Al(OTf)_3$[a]	25	1 mA cm^{-2}, 1 mAh cm^{-2} 200+ h	1 mA cm^{-2}, 1 mAh cm^{-2} 98%, 300 cycles	5	1C, 94.5*	73.2%, 700 cycles*	14
Poly-DOL+TB[a]	-	1 mA cm^{-2}, 0.5 mAh cm^{-2} 800+ h	0.5 mA cm^{-2}, 0.5 mAh cm^{-2} 98.1%, 200+ cycles	-	2C, 126.6*	96.3%, 1200 cycles*	15
PETEA-based CGPE PDAALi- GF-DOL/DME/ LiTFSI[a]	25	1 mA cm^{-2}, 3 mAh cm^{-2} 500+ h	0.5 mA cm^{-2}, 5 mAh cm^{-2} 98.3%, 100 cycles	-	-	-	16

[a] Contains liquid electrolyte.

[b] Composite solid electrolyte.

[*] Values calculated using ImageJ analysis software.

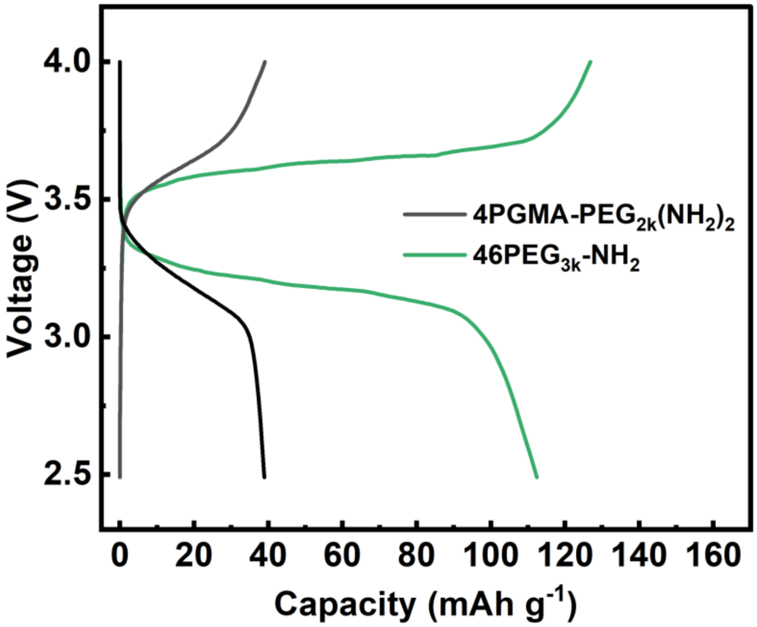

Figure S15. Voltage profile of 1st charge/discharge cycle in represenative 4PGMA-$PEG_{2k}(NH_2)_2$ and $46PEG_{3k}$-NH_2 cells cycled at a rate of 0.33C and 40 °C.

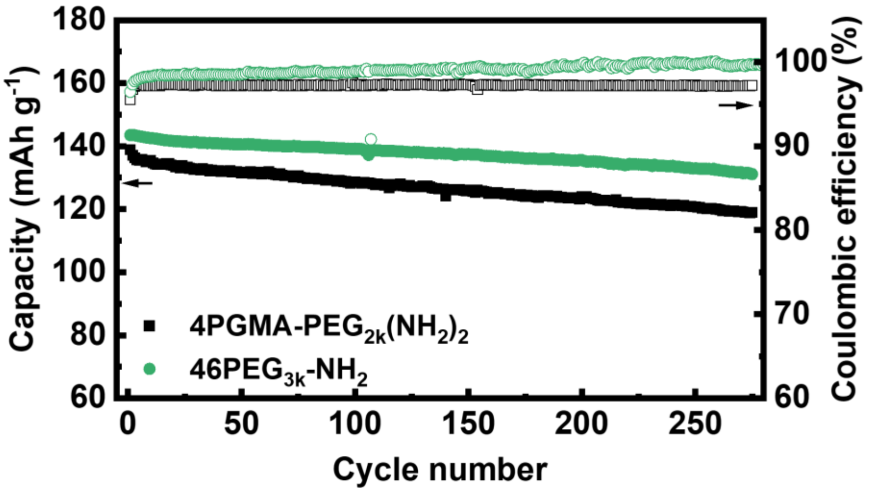

Figure S16. 4PGMA-PEG$_{2k}$(NH$_2$)$_2$ and 46PEG$_{3k}$-NH$_2$ cells cycled at 1C and 90 °C used for EIS measurements prior to and after cycling.

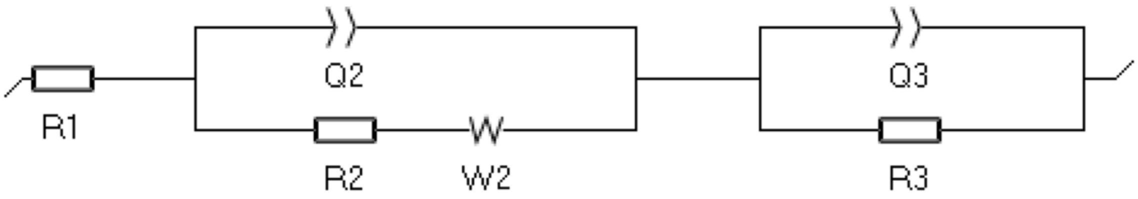

Figure S17. Equivalent circuit model used to fit EIS data of for Li|SPE|$LiFePO_4$ prior to cycling. R_i , Q_i, and W_i represent resistive, constant phase and semi-infinite Warburg elements, respectively. R1, R2 and R3 were taken to represent the bulk electrolyte, cathode-SPE and anode-SPE resistances, respectively.

Table S5. Interfacial and bulk resistances obtained through fitting EIS data for Li|SPE|$LiFePO_4$ cells prior to cycling.

SPE	Bulk resistance (Ω cm^2)	Anode-SPE interfacial resistance (Ω cm^2)	Cathode-SPE interfacial resistance (Ω cm^2)
4PGMA-$PEG_{2k}(NH_2)_2$	18	11	34
46PEG_{3k}-NH_2	9	11	30

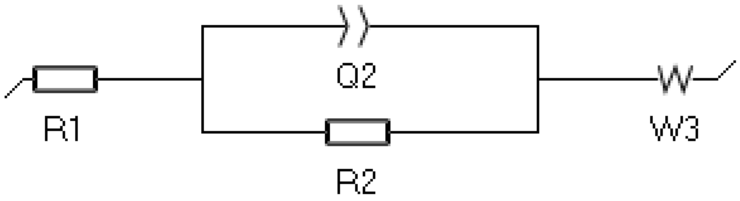

Figure S18. Equivalent circuit model used to fit EIS data of for Li|SPE|$LiFePO_4$ after cycling at 1C. R_i , Q_i, and W_i represent resistive, constant phase and semi-infinite Warburg elements, respectively. R1 & R2 were taken to represent the bulk electrolyte and anode-SPE interfacial resistances, respectively. The W4 semi-infinite Warburg element was introduced to improve the fit in the lower frequency region.

Table S6. Interfacial and bulk resistances obtained through fitting EIS data for Li|SPE|$LiFePO_4$ cells after cycling.

SPE	Bulk resistance (Ω cm^2)	Anode-SPE interfacial resistance (Ω cm^2)
4PGMA-$PEG_{2k}(NH_2)_2$	40	116
46PEG_{3k}-NH_2	12	40

*Corresponding author: Chrisli@drexel.edu, orcid.org/0000-0003-2431-7099

References

(1) Imholt, L.; Dörr, T. S.; Zhang, P.; Ibing, L.; Cekic-Laskovic, I.; Winter, M.; Brunklaus, G. Grafted polyrotaxanes as highly conductive electrolytes for lithium metal batteries. *J. Power Sources* **2019**, *409*, 148-158.
(2) Cao, C.; Li, Y.; Feng, Y.; Peng, C.; Li, Z.; Feng, W. A solid-state single-ion polymer electrolyte with ultrahigh ionic conductivity for dendrite-free lithium metal batteries. *Energy Storage Mater.* **2019**, *19*, 401-407.
(3) Li, X.; Zheng, Y.; Pan, Q.; Li, C. Y. Polymerized Ionic Liquid-Containing Interpenetrating Network Solid Polymer Electrolytes for All-Solid-State Lithium Metal Batteries. *ACS Appl. Mater. Inter.* **2019**, *11* (38), 34904-34912.
(4) Wu, J.; Rao, Z.; Cheng, Z.; Yuan, L.; Li, Z.; Huang, Y. Ultrathin, Flexible Polymer Electrolyte for Cost- Effective Fabrication of All- Solid- State Lithium Metal Batteries. *Adv. Energy Mater.* **2019**, *9* (46), 1902767.
(5) Zheng, Y.; Li, X.; Li, C. Y. A novel de-coupling solid polymer electrolyte via semi-interpenetrating network for lithium metal battery. *Energy Storage Mater.* **2020**, *29*, 42-51.
(6) Li, X.; Zheng, Y.; Duan, Y.; Shang, M.; Niu, J.; Li, C. Y. Designing Comb-Chain Crosslinker-Based Solid Polymer Electrolytes for Additive-Free All-Solid-State Lithium Metal Batteries. *Nano Lett.* **2020**, *20* (9), 6914-6921.
(7) Wang, Z.; Shen, L.; Deng, S.; Cui, P.; Yao, X. 10 μm- Thick High- Strength Solid Polymer Electrolytes with Excellent Interface Compatibility for Flexible All- Solid- State Lithium-Metal Batteries. *Adv. Mater.* **2021**, *33* (25), 2100353.
(8) Ma, Y.; Wan, J.; Yang, Y.; Ye, Y.; Xiao, X.; Boyle, D. T.; Burke, W.; Huang, Z.; Chen, H.; Cui, Y.; Yu, Z.; Oyakhire, S. T.; Cui, Y. Scalable, Ultrathin, and High- Temperature- Resistant Solid Polymer Electrolytes for Energy- Dense Lithium Metal Batteries. *Adv. Energy Mater.* **2022**, *12* (15), 2103720.
(9) Zhang, X.; Wang, S.; Xue, C.; Xin, C.; Lin, Y.; Shen, Y.; Li, L.; Nan, C.-W. Self-Suppression of Lithium Dendrite in All-Solid-State Lithium Metal Batteries with Poly(vinylidene difluoride)-Based Solid Electrolytes. *Adv. Mater.* **2019**, *31* (11), 1806082.
(10) Li, Z.; Xie, H.-X.; Zhang, X.-Y.; Guo, X. In situ thermally polymerized solid composite electrolytes with a broad electrochemical window for all-solid-state lithium metal batteries. *J. Mater. Chem. A* **2020**, *8* (7), 3892-3900, 10.1039/C9TA09969G.
(11) Hu, J.; Lai, C.; Chen, K.; Wu, Q.; Gu, Y.; Wu, C.; Li, C. Dual fluorination of polymer electrolyte and conversion-type cathode for high-capacity all-solid-state lithium metal batteries. *Nat. Commun.* **2022**, *13* (1).
(12) Yao, M.; Ruan, Q.; Yu, T.; Zhang, H.; Zhang, S. Solid polymer electrolyte with in-situ generated fast Li+ conducting network enable high voltage and dendrite-free lithium metal battery. *Energy Storage Mater.* **2022**, *44*, 93-103.
(13) Liu, C.; Wang, J.; Kou, W.; Yang, Z.; Zhai, P.; Liu, Y.; Wu, W.; Wang, J. A flexible, ion-conducting solid electrolyte with vertically bicontinuous transfer channels toward high performance all-solid-state lithium batteries. *Chem. Eng. J.* **2021**, *404*, 126517.
(14) Zhao, Q.; Liu, X.; Stalin, S.; Khan, K.; Archer, L. A. Solid-state polymer electrolytes with in-built fast interfacial transport for secondary lithium batteries. *Nat. Energy* **2019**, *4* (5), 365-373.
(15) Xiang, J.; Zhang, Y.; Zhang, B.; Yuan, L.; Liu, X.; Cheng, Z.; Yang, Y.; Zhang, X.; Li, Z.; Shen, Y.; Jiang, J.; Huang, Y. A flame-retardant polymer electrolyte for high performance

lithium metal batteries with an expanded operation temperature. *Energy Environ. Sci.* **2021**, *14* (6), 3510-3521, 10.1039/D1EE00049G.
(16) Zhou, D.; Tkacheva, A.; Tang, X.; Sun, B.; Shanmukaraj, D.; Li, P.; Zhang, F.; Armand, M.; Wang, G. Stable Conversion Chemistry- Based Lithium Metal Batteries Enabled by Hierarchical Multifunctional Polymer Electrolytes with Near- Single Ion Conduction. *Angew. Chem. Int. Ed.* **2019**, *58* (18), 6001-6006.
